# Analysis of a Serial/Parallel Type of Electromagnetic Actuator

**DOI:** 10.3390/s20102762

**Published:** 2020-05-12

**Authors:** Kenta Takei, Wataru Kitagawa, Takaharu Takeshita, Yoshio Fujimura

**Affiliations:** 1 Department of Electrical and Mechanical Engineering, Nagoya Institute of Technology, Nagoya 466-8555, Japan; kitagawa.wataru@nitech.ac.jp (W.K.); take@nitech.ac.jp (T.T.); 2 Wako Engineering Co., Ltd., Gifu 500-8234, Japan; y-fujimura@wako-engineering.co.jp

**Keywords:** electromagnetic actuator, finite element method, electromagnetic field analysis, mapping

## Abstract

This paper describes the design and analysis of a small-sized and high thrust electromagnetic actuator. The proposed actuator is supposed to be used for application control of the hotmelt adhesive. The hotmelt has different characteristics for each material and the electromagnetic actuator is required variable characteristics. However, the problem seems to lie in the fact that it is necessary to remake another mold again to change the characteristics of the conventional electromagnetic actuator. Therefore, this paper presents small-sized electromagnetic actuator called a basic model that can stack it in the axial direction or in the radial direction. As the analysis comparison at the same size, the characteristics of conventional two serial model which stack two basic models in the axial direction and proposed three serial models have been compared by three-dimensional finite element method. In the proposed model, characteristics have been improved by reducing the core volume and increasing the number of stacks in the basic model from the viewpoint of magnetic flux density. In addition, various electromagnetic actuators that stack basic models in the axial direction or in the radial direction have been analyzed. The analysis results have been clearly shown as characteristics mapping and it has indicated that the proposed electromagnetic actuator can be constructed easily by stacking the basic model.

## 1. Introduction

The proposed actuator is considered for use in the control of applying the hotmelt. The hotmelt is a thermoplastic resin which is solid at normal temperature. Hence, the hotmelt becomes liquefied when it is heated, and it is solidified when it becomes cooled by the air [1,2]. By this characteristic, it is used for adhering objects such as packaging of cardboard and non-woven fabric, robotic structure [3,4]. It does not contain organic solvents, therefore, it is friendly to humans and the environment. Moreover, it enables us to improve productivity due to its quick-drying, and the optimal adhesion can be chosen for each adhesion object. Thus the hotmelt is used at many factory lines [5]. The application method of the hotmelt is widely varied. Therefore, the applicator for control of applying the hotmelt is required for the precise adjustment of adhesion and a high response to applying it with complicated methods.

Nowadays the pneumatic actuator is used for the hotmelt applicator. The pneumatic actuator is a simple structure and it generates high thrust easily because it can accumulate compressed air in the tank. However, the response delay of the pneumatic actuator is large and it is difficult to control quickly. In addition, large equipment is needed large such as an air compressor and pipeline. Moreover, it takes time to compress the air in the cylinder, and it is required periodic maintenance to fix the worn sliding parts.

On the other hand, the electromagnetic actuator has a good response. Moreover, it can minimize equipment because it can be driven by the power supply [6]. However the entire equipment becomes hot due to heating the hotmelt. The permanent magnet included in the electromagnetic actuator has a magnetic characteristic depending on the temperature. Therefore when the permanent magnet becomes hot, the thrust and the response of the electromagnetic actuator are decreased [7,8,9,10]. For this reason, we needed to design the electromagnetic actuator that meets the required performance and to analyze the characteristic of the proposed actuator. So far the small-sized and high thrust electromagnetic actuator is proposed [11,12,13].

In this study, the electromagnetic actuator that can change characteristics has been devised because the hotmelt adhesives have various characteristics for each material [14,15,16,17,18]. As a result, the small-sized electromagnetic actuator called basic model has been designed. The basic model can be changed the characteristics by stacking it. This paper presents the small-sized electromagnetic actuator that can be stacked in the axial direction or in the radial direction. In addition, the conventional basic model could reduce the core volume from the viewpoint of magnetic flux density, and the proposed three serial models could have been constructed with the same size as the two-serial model. Therefore, the characteristics of the conventional two-serial model and the proposed a three-serial model have been compared. As a result, it has been confirmed that the characteristics have been improved in the proposed model. In addition, the characteristics of the serial model and the parallel model, which have been stacked in the basic model, and the various types of electromagnetic actuators combined have been analyzed. The analysis results have been clearly shown as characteristics mapping and it has indicated that the proposed electromagnetic actuator can be constructed easily by stacking the basic model.

Finally, future works are described in this study. First, the analysis should be taken into account the high-temperature environment around the electromagnetic actuator [19]. This paper does not consider the high-temperature environment for melting the hotmelt, but considers the characteristics and temperature rise of the electromagnetic actuator on the high-temperature environment. Second, it is important to examine the validity of the analysis by making the experimental machine and comparing experimental results with simulation results.

## 2. Model Structure and Operating Principle

### 2.1. Basic Model Structure

**Figure 1:**
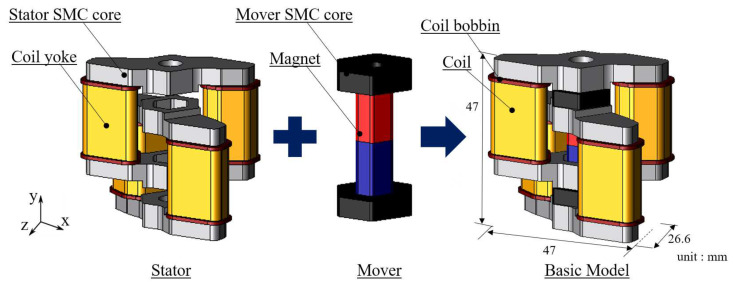
Structure of basic model.

The basic model is composed of a stator and a mover, a plunger needle, a cover. Figure 1 shows the structure of the stator and the mover, the basic model. In this paper, the plunger needle and the cover aren’t considered.

For the stator, stator cores are arranged in the chain pattern. Coil yokes are arranged between the stator cores. Coil bobbins are arranged outside of the coil yoke, and coils are wound on the coil bobbin. As for the mover, the permanent magnet is arranged between mover cores. The materials of the stator core and mover core, coil yoke are soft magnetic composite (SMC) core [20,21].

In the conventional model, the stator core can be cut because it has a margin from the viewpoint of the magnetic flux density. Therefore, the stator core is cut and the coil height has been shortened in the proposed model. As a result, the proposed model with the almost same size as the conventional model can stack three basic models.

### 2.2. Operating Principle

**Figure 2:**
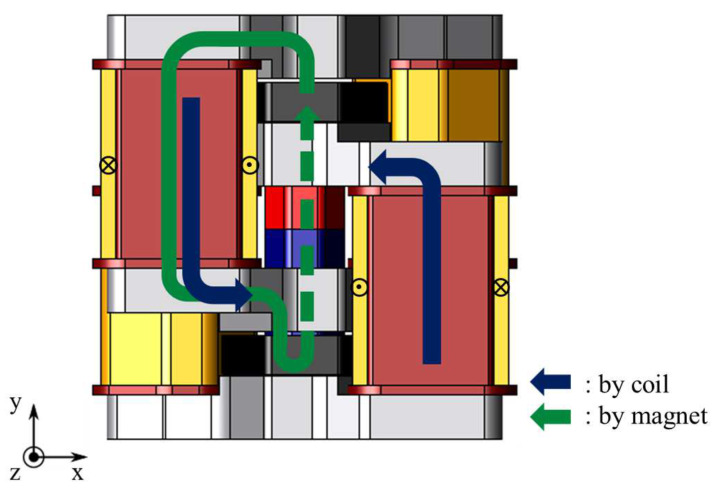
Operating principle.

Figure 2 shows the magnetic flux flow. The stator cores are magnetized by the magnetic field generated by the electromagnet. Moreover the mover cores are magnetized by the permanent magnet. The electromagnetic force is generated between the stator core and the mover core. Therefore, an upward thrust works to the mover. On the other hand, when the magnetic poles of the stator cores are reversed by changing the direction of flowing current, a downward thrust works to the mover. The hotmelt applicator has an adhesive discharge port in the lower part of the proposed actuator. The port is cut off when the upward thrust works, and the port is open when the downward thrust works. Therefore, the proposed actuator is required the maximum thrust value 132 N to realize the complicated adhesion methods.

## 3. Expansion to Serial/Parallel Type

**Figure 3:**
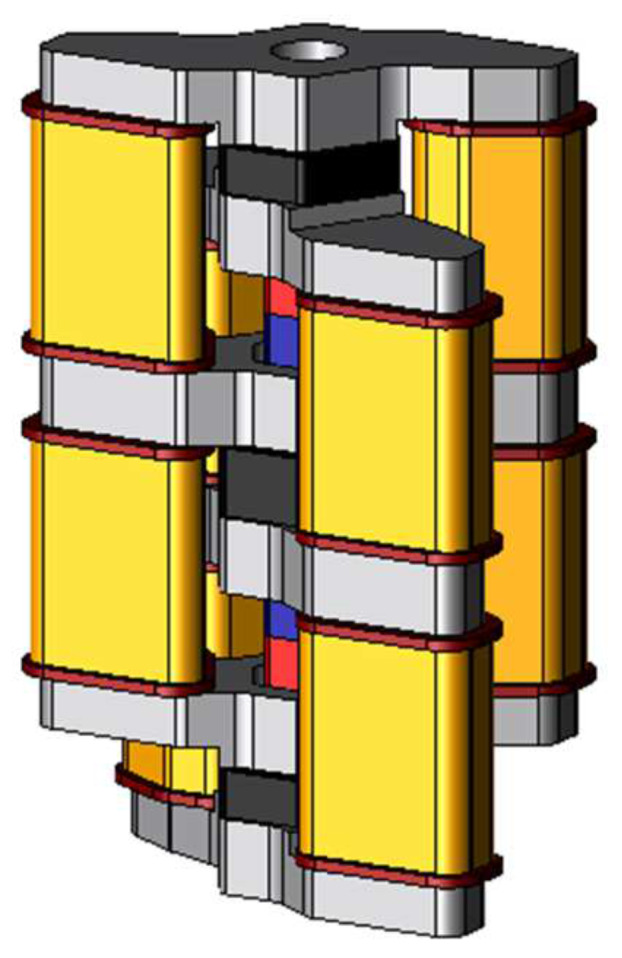
Structure of the two-serial model.

**Figure 4:**
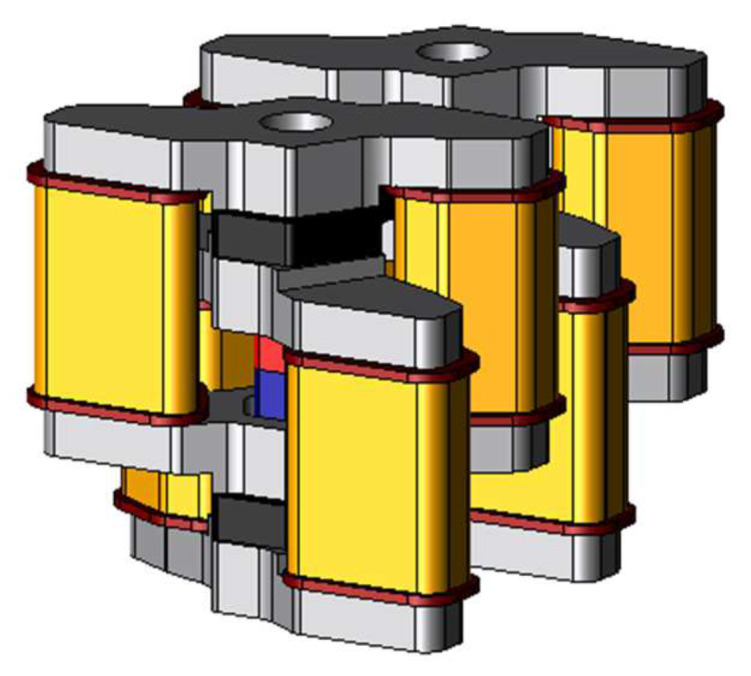
Structure of the two-parallel model.

The major feature of the proposed model is its structure. The structure of the basic model is a chain pattern and the proposed actuator can generate high thrust easily by stacking the basic models or arranging them. Figure 3 shows the two-serial models stacked with two basic models. As for the stator, the stator cores are joined by the coil yoke similar to the basic model. As for the mover, two permanent magnets are arranged between three mover cores. The permanent magnets are magnetized mutually reverse directions. Hence the magnetic flux of the permanent magnets flows through different magnetic paths, and it can make suppress the magnetic saturation on the coil yoke. Figure 4 shows the two-parallel model arranged as two basic models. The structure of the stator is the same as the basic model. As for the mover, mover-connection is arranged between adjacent the mover cores.

## 4. Analysis Condition

**Figure 5:**
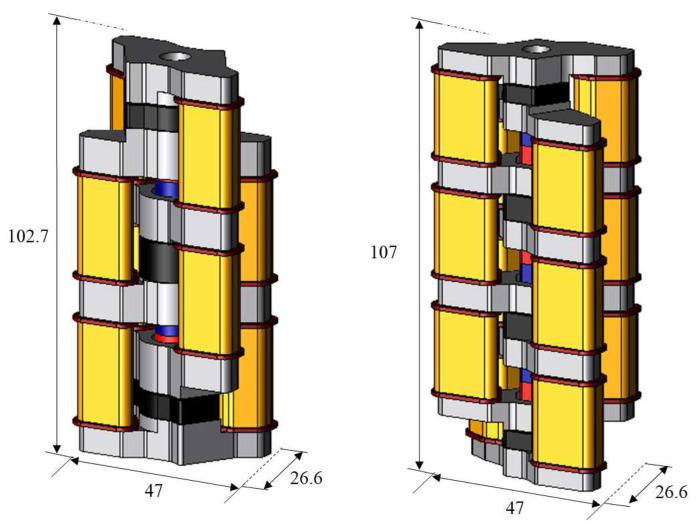
Structure of the conventional model and proposed model.

**Figure 6:**
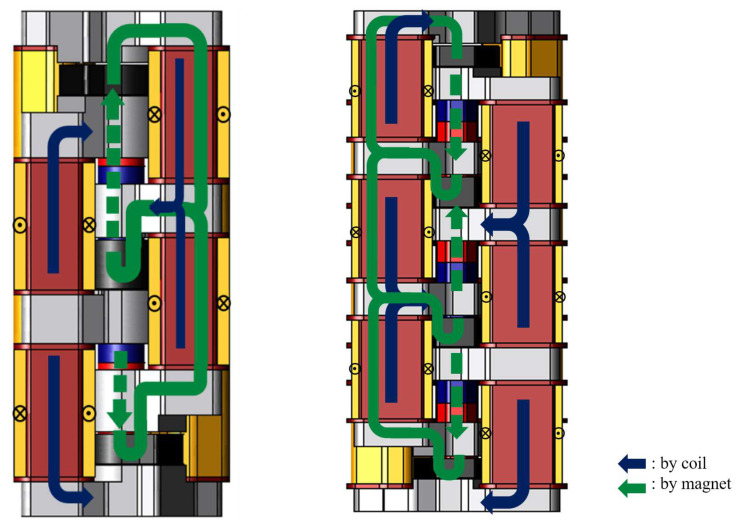
Flux flow of the conventional model and proposed model.

First, the characteristics of the conventional model and the proposed model are compared. In this paper, the proposed electromagnetic actuator is analyzed using JMAG, a three-dimensional finite element method analysis software. Figure 5 shows the structure of the conventional model and the proposed model and Figure 6 shows the flux flow of the conventional model and the proposed model. The conventional model can reduce the stator core volume from the viewpoint of magnetic flux density. Therefore, the stator core volume has been reduced by 46% in the y-axis direction, and the height of coil and coil yoke has been shortened 30 mm to 25 mm and 26 mm to 21 mm. As a result, the shape has been changed so that three basic modes can be stacked at the same height as the conventional two-serial model. The serial type and parallel type of the proposed model are analyzed. Table 1 shows the analysis condition. The amplitude of the current is 1 A, the current rise time is 0.125 ms, and its frequency is 500 Hz. In the conventional model, the number of coil turn is 526 turn. On the other hand, it has 516 turns in the proposed model because the coil height has been shortened.

**Figure 7:**
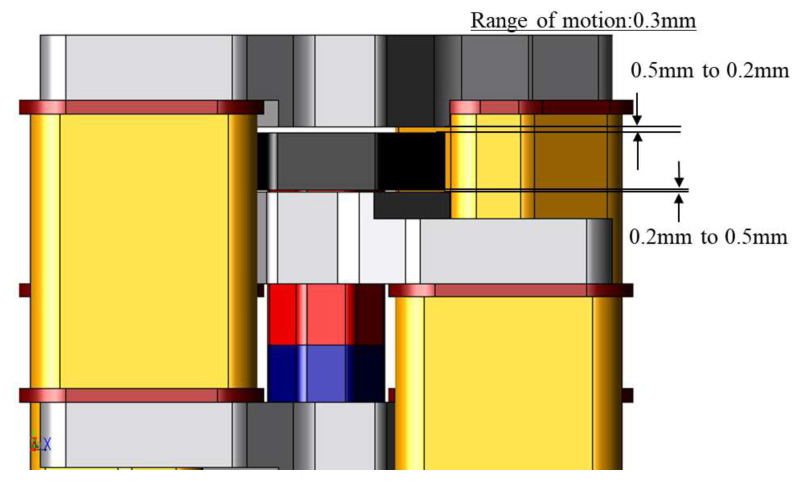
Range of motion.

Figure 7 shows the range of motion of the proposed actuator. The range is 0.3 mm. The mover goes to the descending stroke end and returns to the ascending stroke end. When the mover is at the descending stroke end, the gap between the mover core and the descending side stator core is 0.2 mm. When the mover is at the ascending stroke end, the gap between the mover core and the ascending side stator core is 0.2 mm.

The following equation represents the equation of motion for considering the motion of mover.

(1) $$F_{\left(y\right)}=m\frac{d^{2}y}{dt^{2}}+\gamma \frac{dy}{dt}+ky,$$

where the parameter *m* is the mass, γ is the viscous damping, *k* is the spring constant, *y* is displacement. In this paper, γ and *k* are zero for the sake of the analysis on no load condition.

## 5. Analysis Result

### 5.1. Comparison of Conventional Model and Proposed Model

**Figure 8:**
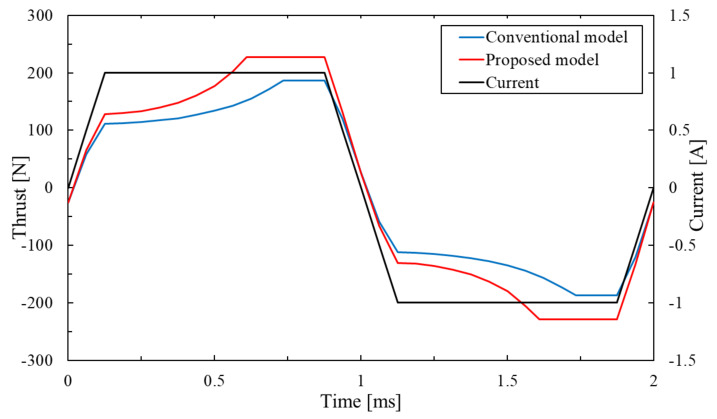
Thrust waveforms of the conventional model and proposed model.

**Figure 9:**
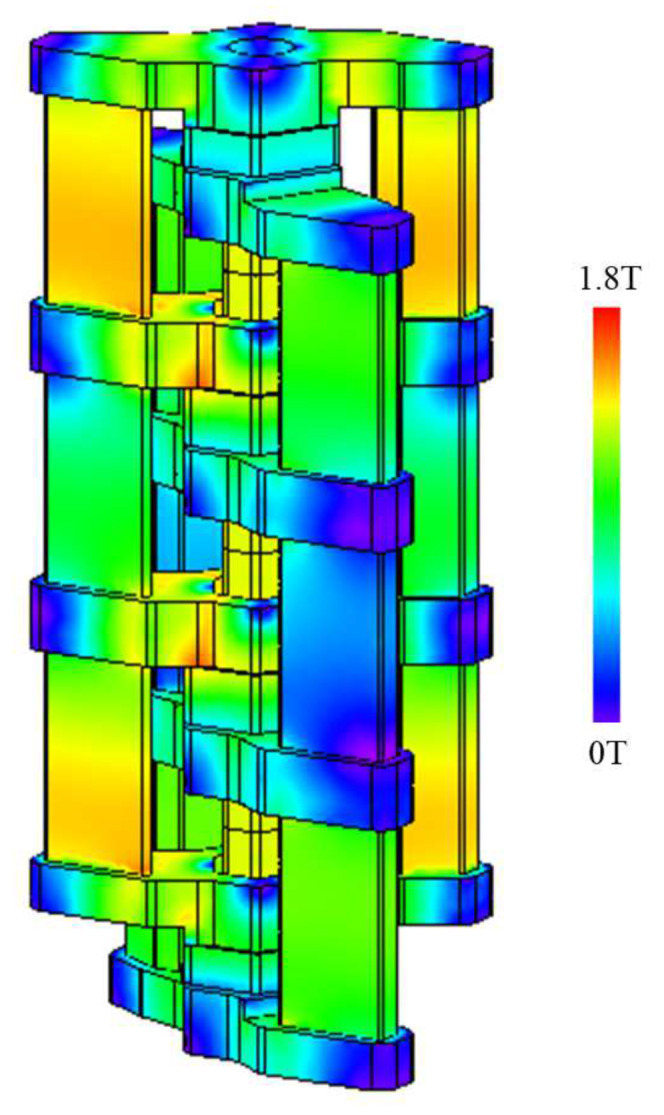
Magnatic flux density distribution.

Figure 8 shows the thrust waveforms of the conventional model and the proposed model. The maximal thrust value of the conventional model is 186.3 N. In contrast, the maximal thrust value of the proposed model is 226.8 N. As a result, the proposed model can improve the maximal thrust value by 21.7% than the conventional model. The reason is that the change in the proposed model from the two-serial model to the three-serial model increases the magnetic flux by the coil and permanent magnet. The coil yoke is structurally likely to have high magnetic flux density in the proposed model. Figure 9 shows that the magnetic flux is not saturated in the proposed model. In the ideal magnetic flux path of the three-serial model shown in Figure 6, the magnetic flux caused by the permanent magnet forms the magnetic circuit only on the left side but some magnetic flux also flows to the right side because the gap between the mover core and the stator core is short. The magnetic flux does not flow so much in the central coil yoke but flows in the upper and lower coil yokes because the proposed actuator has been not well balanced yet. The future work is to improve this problem on the proposed model.

**Figure 10:**
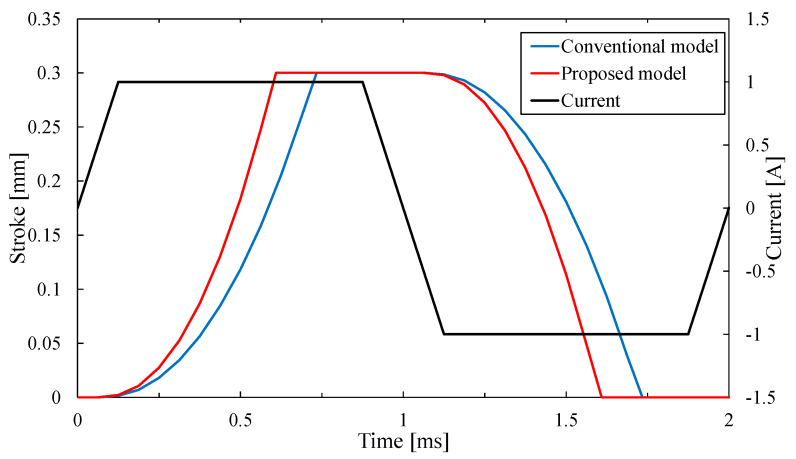
Response waveforms of the conventional model and proposed model.

Figure 10 shows the response waveforms of the conventional model and the proposed model. In Figure 10, the mover starts to ascend from the descending stroke end after passing current and reaches the ascending stroke end in about 0.73 ms on the conventional model. In contrast, the time is about 0.61 ms on the proposed model and the proposed model can improve the response by 16.4% than the conventional model.

**Figure 11:**
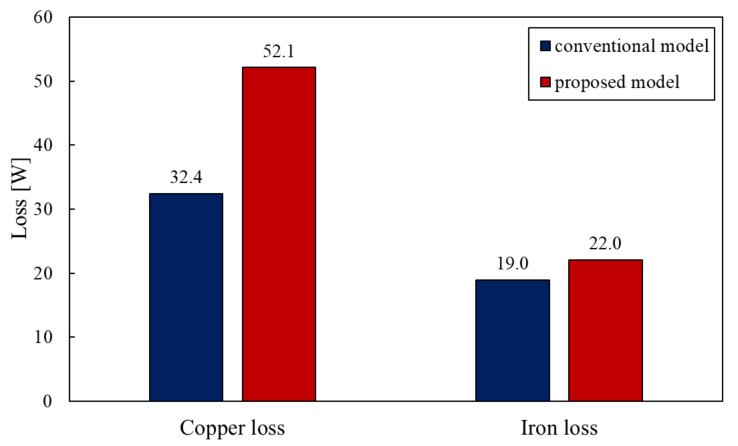
Loss of the conventional model and proposed model.

**Figure 12:**
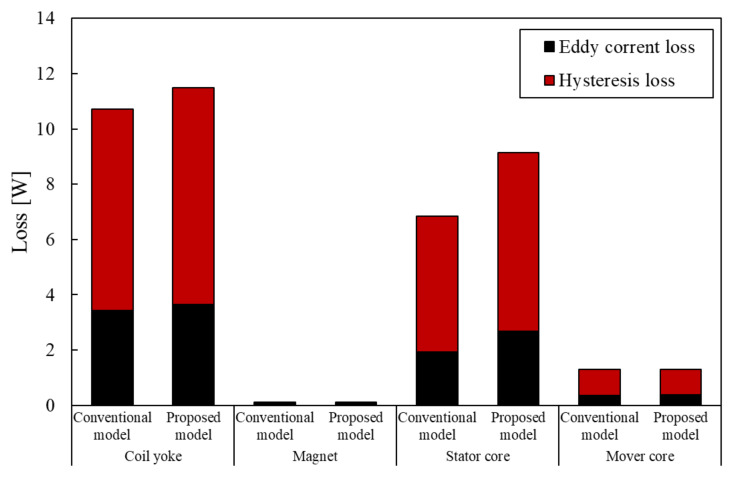
Breakdown of Irion Loss.

Figure 11 shows the loss of the conventional model and the proposed model and Figure 12 shows the breakdown of the iron loss. In these figures, the proposed model has more loss than the conventional model. It is for this reason that the number of coils has increased and the magnetic flux density of the stator core has got highly by reduction of the stator core. It is necessary to conduct thermal analysis to show how heat from this loss affects the actuator.

According to the analysis comparison, the proposed model is effective because it can obtain higher response and thrust than the conventional model as the same size. However, the proposed model should be concerned with efficiency because it has more loss than the conventional model. In the future, we plan to indicate the proposed model is effective by establishing the drive circuit and considering it comprehensively including efficiency.

### 5.2. Characteristics of Serial/Parallel type

**Figure 13:**
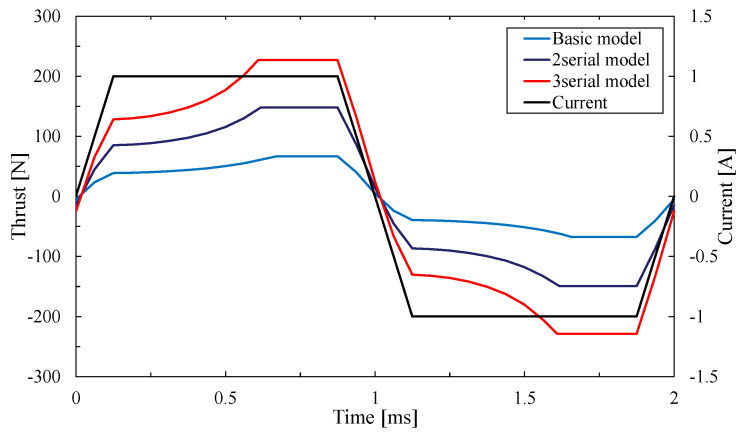
Thrust waveform of serial type.

**Figure 14:**
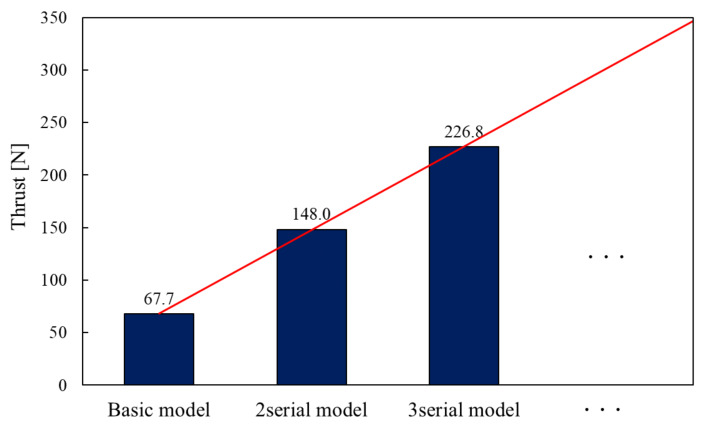
Maximal thrust of the serial type.

The thrust and response of the serial type are considered by the result comparison of the basic model, two-serial type and three-serial type of the proposed model. Figure 13 shows the thrust waveform of each model and Figure 14 shows the maximal thrust value of each model.

As a result, their maximal thrust value is 67.7 N and 150.2 N, 230.3 N. The basic model only cannot exceed the required thrust value. However, two-serial types and three-serial types of the proposed model can exceed it. The serial type of the proposed model can generate more than several times thrust by stacking the basic models. The reason is that the central mover core is arranged in the serial type. The magnetic flux can be used efficiently on the central mover core. Hence the larger magnetic force is generated between the central mover core and stator core.

In addition, it is defined by thrust density that the proposed actuator is small-sized. The volume of the basic model was 58,759 ${\mathrm{mm}}^{3}$, and the thrust density of the basic model was calculated as 0.00113 $N/{\mathrm{mm}}^{3}$. According to the other electromagnetic machines, it is proved that the proposed actuator with high thrust density is small [22,23].

**Figure 15:**
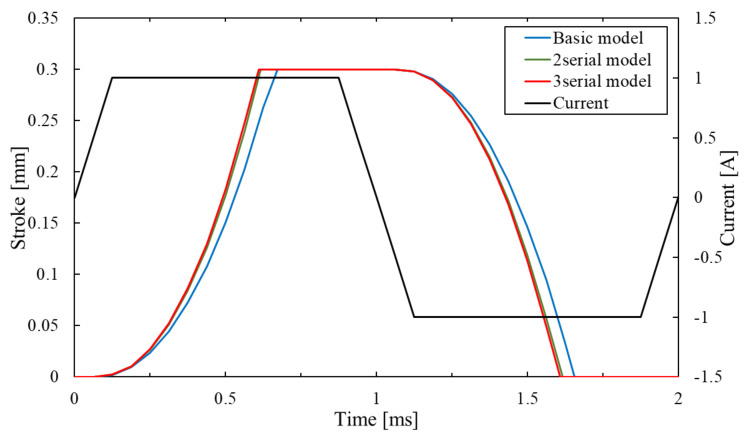
Response waveform of a serial type.

Figure 15 shows the response waveform of the serial type of the proposed model. The stroke time of the mover is about 0.67 ms on the basic model. In contrast, the time of the two-serial type and the three-serial type of the proposed model is about 0.62 ms and 0.61 ms. It is considered that the response of the serial type is better because it can obtain higher thrust.

**Figure 16:**
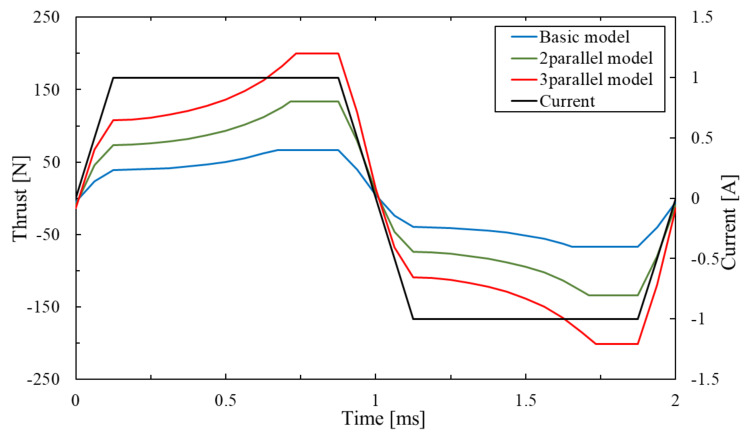
Thrust waveform of parallel type.

**Figure 17:**
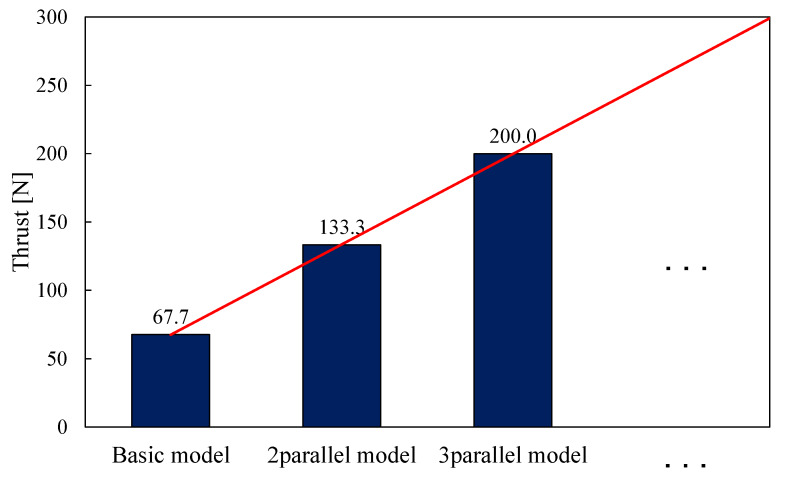
Maximal thrust of parallel type.

Similarly, the thrust and response of the parallel type are considered by the comparison of analysis of the basic model, two-parallel type and three-parallel type of the proposed model. Figure 16 shows the thrust waveform of each model and Figure 17 shows the maximal thrust value of each model.

**Figure 18:**
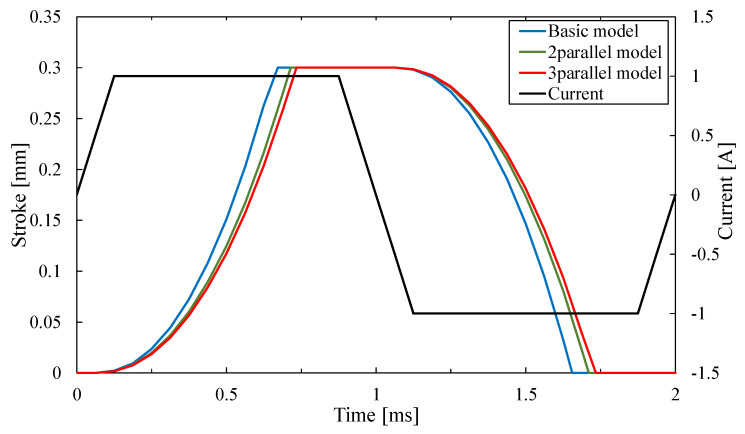
Response waveform of parallel type.

As a result, their maximal thrust value is 67.7 N and 135.2 N, 202.8 N. The two-parallel type and three-parallel type of the proposed model can exceed the required thrust value. The parallel type of the proposed model can generate several times thrust by arranging the basic models. Figure 18 shows the response waveform of the parallel type. The stroke times of two-parallel type and three-parallel type of the proposed model were 0.71 ms and 0.73 ms. The response of the parallel type is worse than the basic model because the weight of the mover is increased by arranging the mover-connection. In this study, the SMC core is used for the mover-connection. However it is confirmed that magnetic flux does not pass through it. Therefore, we will consider using lighter materials such as resin.

**Figure 19:**
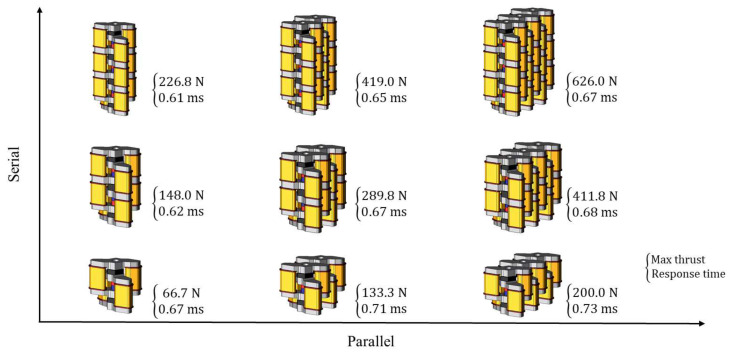
Application of the basic model.

Figure 19 shows the analysis result of the various shapes of the proposed model all the way from the basic model to three-serial three-parallel type. The merit of the proposed model is that it can obtain high thrust for various applications not just the hotmelt application by stacking the basic models or arranging them.

## 6. Conclusions

This paper describes how the conventional model has been improved and the proposed model stacking three basic models as the same size as the conventional model stacking two basic models has been designed. The conventional model and the proposed model has been analyzed by the three-dimensional finite element method and compared the characteristics. As a result, it is revealed that the proposed model obtains the maximal thrust value 21.7% greater than the conventional model and has a response 16.4% greater. The proposed model has obtained higher thrust and response than the conventional model. However, the loss of the proposed model has been increased compared to the conventional model. Therefore, it should be comprehensively considered including the efficiency.

This paper presents the serial type and parallel type of the proposed model. The proposed model can generate high thrust by stacking the basic models in the axial direction or arranging them in the radial direction. The serial type and parallel type of the proposed model have been analyzed and compared to the characteristics. As a result, both of the serial type and parallel type has generated more than several times the thrust of the basic model.

Finally, future works are described in this study. First, the analysis should have taken into account the high-temperature environment around the electromagnetic actuator. This paper does not consider the high-temperature environment for melting the hotmelt. Actually, the high-temperature field might affect the electromagnetic actuator, and the characteristics of the electromagnetic actuator might be decreased. Therefore, it should be considered the characteristics and temperature rise of the electromagnetic actuator in the high-temperature field. Second, it should be considered the experiment with an experimental machine based on the proposed actuator model and the simulation results should be shown to be valid.

## Figures and Tables

**Table 1:** Specifications.

	Conventional Model	Proposed Model
Number of coil turn	526	516
Gap length [mm]	0.2–0.5	0.2–0.5
Material of magnet	NF45UH	NF45UH
Residual magnetic flux density [T]	1.3	1.3
Material of core	FMCM-HB1	FMCM-HB1
Saturation magnetic flux density [T]	1.8	1.8
